# Contribution of Palmitic Acid to Epidermal Morphogenesis and Lipid Barrier Formation in Human Skin Equivalents

**DOI:** 10.3390/ijms20236069

**Published:** 2019-12-02

**Authors:** Arnout Mieremet, Richard Helder, Andreea Nadaban, Gert Gooris, Walter Boiten, Abdoelwaheb El Ghalbzouri, Joke A. Bouwstra

**Affiliations:** 1 Department of Dermatology, Leiden University Medical Center, 2333 ZA Leiden, The Netherlands; a.mieremet@lumc.nl (A.M.); a.e.l.ghalbzouri@lumc.nl (A.E.G.); 2 Division of BioTherapeutics, LACDR, Leiden University, 2333 CC Leiden, The Netherlands; r.w.j.helder@lacdr.leidenuniv.nl (R.H.);

**Keywords:** primary cell culture, artificial skin, bioengineering, palmitic acid, free fatty acid, ceramide, lipid metabolism, mass spectrometry

## Abstract

The outermost barrier layer of the skin is the stratum corneum (SC), which consists of corneocytes embedded in a lipid matrix. Biosynthesis of barrier lipids occurs de novo in the epidermis or is performed with externally derived lipids. Hence, in vitro developed human skin equivalents (HSEs) are developed with culture medium that is supplemented with free fatty acids (FFAs). Nevertheless, the lipid barrier formation in HSEs remains altered compared to native human skin (NHS). The aim of this study is to decipher the role of medium supplemented saturated FFA palmitic acid (PA) on morphogenesis and lipid barrier formation in HSEs. Therefore, HSEs were developed with 100% (25 μM), 10%, or 1% PA. In HSEs supplemented with reduced PA level, the early differentiation was delayed and epidermal activation was increased. Nevertheless, a similar SC lipid composition in all HSEs was detected. Additionally, the lipid organization was comparable for lamellar and lateral organization, irrespective of PA concentration. As compared to NHS, the level of monounsaturated lipids was increased and the FFA to ceramide ratio was drastically reduced in HSEs. This study describes the crucial role of PA in epidermal morphogenesis and elucidates the role of PA in lipid barrier formation of HSEs.

## 1. Introduction

The human skin protects the body from desiccation and environmental challenges through the establishment of a multilayered barrier system. The main physical barrier is formed by the outermost layer of the epidermis, which is the stratum corneum (SC). This SC consists of corneocytes embedded in a lipid matrix (Supplementary Figure S1a–c). The lipid matrix forms the only continuous penetration pathway through the SC. Therefore, it is crucial for the functionality of the barrier.

Sophisticated in vitro tools which resemble native human skin (NHS) to a high extent are three-dimensional human skin equivalents (HSEs). Therefore, these are widely applied in preclinical screenings and for research purposes to increase understanding of skin biology and epidermal barrier formation in healthy and diseased phenotypes [1,2,3,4,5,6]. HSEs mimic NHS in morphologic appearance, including the presence of distinguishable epidermal layers and formation of the SC [7,8,9]. Characterization of the barrier formation in HSEs revealed similarities as well as differences compared to the barrier formation of NHS. Similarities in the SC lipid matrix between HSEs and NHS include the presence of the main lipid classes and subclasses thereof, and the presence of distinctive lipid lamellae in the intercorneocyte space [7,8,10]. However, differences were reported regarding the lipid composition, such as an increased level of monounsaturated lipids, a reduced chain length of free fatty acids (FFAs) and ceramides (CERs), and a different CER subclass profile [10,11]. Changes in lipid composition coincides with a reduction in the length of the repeat distance of the long periodicity phase (LPP), as well as a lack in the formation of the short periodicity phase (SPP) (Supplementary Figure S1d,e) [7,12]. Also, the formation of a predominant hexagonal lateral organization was observed in HSEs, in contrast to a predominant orthorhombic lateral organization in NHS (Supplementary Figure S1f) [8]. These compositional and organizational differences directly contribute to an impaired skin barrier function, as compound penetration occurs at an elevated rate [4,5,7]. Although the altered composition of CERs in the SC of HSE is relatively well described [7,10,11,13], there is still a need for more detailed characterization of the composition of FFA in the SC of HSEs. 

For the formation of the epidermal barrier, FFAs are essential for numerous processes. Most prominent are to serve as building blocks for more complex lipids (e.g., CERs) of the lipid barrier and to contribute to the structure of the lipid matrix [14,15,16]. FFAs are mainly synthesized de novo in the skin, although these could also be taken up from the systemic circulation originating from dietary resources [14,17]. Both are important, since the epidermis can switch between local to systemic sources for lipid barrier repair [17,18]. To improve the barrier formation in HSEs, it is essential to better comprehend the in vitro lipid biosynthesis pathways.

To mimic the systemic availability of lipids, the culture medium of HSEs is supplemented with a mixture of FFAs during submerged and air-exposed phases. This mixture consists of the essential FFA linoleic acid (LA; C18:2(ω-6)), the conditionally essential FFA arachidonic acid (AA; C20:4(ω-6)), and the saturated FFA (saFFA) palmitic acid (PA; C16:0). These exogenous FFAs are shown to be taken up and processed in the epidermis of HSEs to become part of the SC lipid barrier [19,20,21]. Several studies have been performed to optimize the timepoint of supplementation, concentration, and ratio of FFAs during the development of HSEs [19,20,22]. These showed that supplementation with FFAs was crucial to induce the formation of lamellar bodies [22], although a fourfold increase in either PA level or in total FFA mixture in the medium during generation of HSEs did not modulate the FFA composition in SC [19]. However, too high concentrations of supplemented saFFAs could lead to lipotoxic responses [23,24]. This would result in an elevated stearoyl-CoA desaturase-1 (SCD-1) expression and a higher level of monounsaturated lipids [25], which are also observed in HSEs [11]. However, LA and AA are not involved in a saFFA-induced lipotoxic response, which is mediated by increased expression of SCD-1 [26]. Considering this, it is of interest to elucidate the role of supplementation levels of PA during generation of HSEs.

In this study, we aim to decipher the effect of PA supplementation on the epidermal morphogenesis and barrier formation of HSEs and to obtain more insights in the presence of FFAs in the SC of HSEs. To this end, we supplemented the medium with reduced PA concentrations and studied the dermal and epidermal morphogenesis and lipid barrier formation of full thickness models (FTMs) and NHS. Herein, we report that the original PA supplementation concentration is most optimal for the formation of a well-ordered epidermal morphogenesis. Additionally, we observed a considerable reduction in the absolute amount of FFAs in the SC of FTMs as compared to that of NHS, irrespective of the PA concentration.

## 2. Results

### 2.1. FTMs Generated with Various PA Levels Displayed Similar Epidermal Architecture

**Figure 1:**
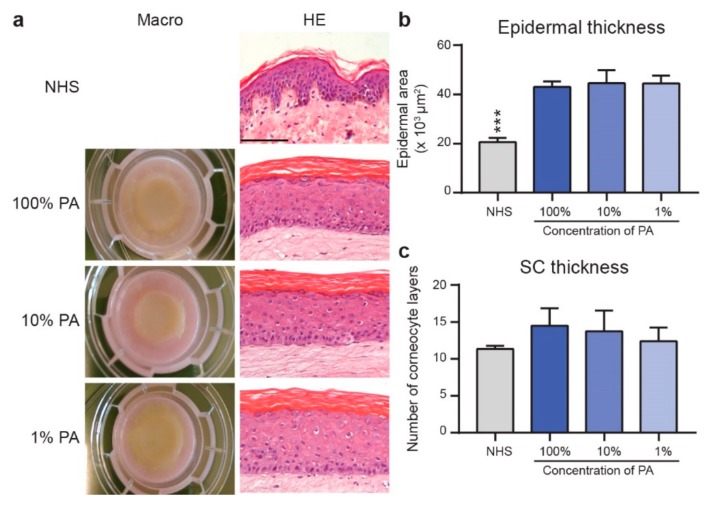
Generation of full thickness models (FTMs) supplemented with various palmitic acid (PA) levels. (**a**) Macro and micro anatomy of native human skin (NHS) and indicated FTMs. Histology examined after hematoxylin and eosin (HE) staining. Scale bar represents 100 μm. (**b**) Quantified epidermal thickness of NHS and indicated FTMs. (**c**) Number of corneocyte layers reflects the stratum corneum (SC) thickness. Data represents mean + SD, *n* = 4, *** indicates *p* < 0.001.

FTMs were developed with 100% (FTM_100%PA_), 10% (FTM_10%PA_), or 1% (FTM_1%PA_) of the original medium PA concentration (25 µM) and were compared to NHS (Figure 1a). This revealed a similar appearance in all conditions tested. Quantification of the thickness of the viable epidermis and number of corneocyte layers revealed an equal epidermal thickness and a similar number of corneocyte layers in FTMs, irrespective of PA level (Figure 1b,c). As compared to NHS, the FTMs lack the epidermal rete ridges and have a thicker viable epidermis. The number of corneocyte layers was similar, despite the lack in exfoliation in FTMs. This indicates that FTMs supplemented with reduced level of PA contain a well-ordered epidermal architecture.

### 2.2. Strong Reduction in PA Supplementation Level Compromised the Epidermal Morphogenesis

**Figure 2:**
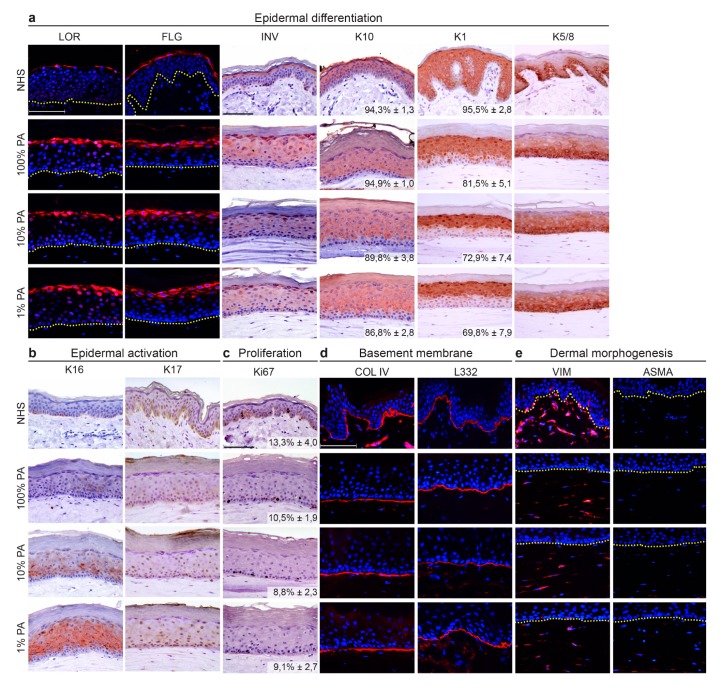
Morphogenesis of FTMs supplemented with different PA levels. Expression of protein biomarkers in NHS and FTMs of (**a**) late and terminal differentiation (loricrin, filaggrin, and involucrin), early differentiation (keratin 10 and 1), and basal layer (keratin 5/8). The percentage of K10 and K1 positive area in the suprabasal epidermis is provided (mean ± SD, *n* = 4). (**b**) Epidermal activation (keratin 16 and keratin 17), (**c**) proliferation (Ki67) with indicated proliferation index (mean ± SD, *n* = 4), (**d**) dermal–epidermal junction (collagen type IV and laminin 332), (**e**) fibroblasts distribution (vimentin), and fibroblasts stress signaling (alpha smooth muscle actin) biomarker protein expression. Nuclei are counterstained blue using hematoxylin or DAPI, yellow dotted line indicates dermal–epidermal junction. Scale bar indicates 100 μm.

Assessments of epidermal and dermal morphogenesis were performed using protein biomarkers. The late differentiation program was unaffected by reduction in PA (loricrin, filaggrin, involucrin) (Figure 2a). As compared to NHS, involucrin was more expressed in the spinous layer, while filaggrin and loricrin were equally localized. Early differentiation, which indicates the transition of keratinocytes from the basal cell layer to the spinous cell layer, was delayed in HSEs developed with reduced PA levels, most severe at 1% indicated by keratin 10 (K10) and 1 (K1) expression. This was confirmed after quantification of the K10 or K1 positive area in the suprabasal viable epidermis, which was lowest in FTM_1%PA_. Lower epidermal layer biomarker K5/8 was expressed in two epidermal segments in FTM_100%PA_, whereas it was diffuse expressed throughout the epidermis of FTM_10%PA_ and FTM_1%PA_. As compared to NHS, expression of the K5/8 proteins is detected in more suprabasal epidermal layers. Epidermal activation of the viable epidermis was found to be directly affected by PA supplementation levels (Figure 2b). Moderate K16 expression was detected in FTM_10%PA_, whereas strong K16 expression was detected in FTM_1%PA_. Nevertheless, K17 remained absent in all conditions. As compared to NHS, epidermal activation was only present in vitro. Another characteristic of epidermal morphogenesis is the proliferation of the viable epidermis. Biomarker Ki67 remained expressed only at the basal layer and the proliferation index was comparable in all conditions (Figure 2c). The viable epidermis is connected to the dermis via the basement membrane, which was generated in vitro in a similar proportion at all exogenous PA levels (Figure 2d). As PA is a lipid which could also be bioactive in the dermis [15,27], dermal morphogenesis was examined for fibroblast distribution and fibroblasts subpopulations with focus on myofibroblasts (Figure 2e). Both were similar regardless of PA levels, although fibroblast distribution in FTMs was more continuous and not divided in a papillary and reticular dermal zone as observed in NHS.

### 2.3. Supplementation of FTMs with Various PA Levels Resulted in an Equal Composition of FFA in the SC 

**Figure 3:**
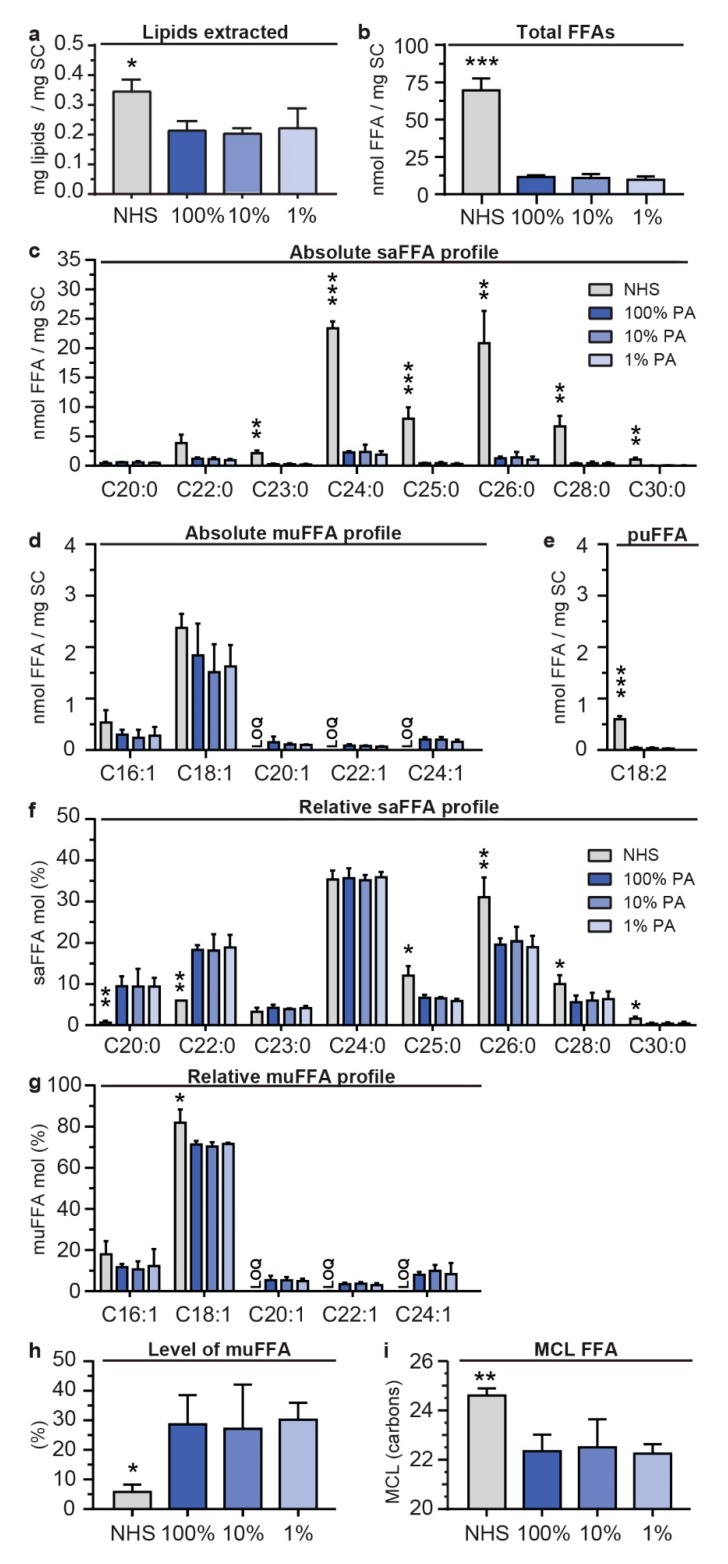
Free fatty acid (FFA) composition in the SC of NHS and in FTM supplemented with different PA levels. (**a**) Extracted lipids were plotted per mg SC of NHS and the FTMs supplemented with 100%, 10%, or 1% PA. (**b**) Absolute amount of FFAs in the SC of NHS and of indicated FTMs. (**c**) Bar diagram of the saturated FFA (saFFA) profile as presented in absolute amount. (**d**) Bar diagram of the monounsaturated FFA (muFFA) profile as presented as absolute amount. (**e**) Bar diagram of the puFFA C18:2 per mg SC. (**f**) Relative amount of saFFAs in the SC of NHS and FTMs. (**g**) Relative amount of muFFAs in the SC of NHS and FTMs. (**h**) The level of muFFAs as percentage of total FFAs. All data for NHS, FTM_100%PA_, FTM_10%PA_, and FTM_1%PA_. (**i**) Bar diagram of the MCL of total FFAs. Data represents mean + SD, *n* = 3. LOQ indicates below limit of quantification. Significance indicated by * *p* < 0.05, ** *p* < 0.01, *** *p* < 0.001.

We then evaluated the lipid barrier formation in the FTMs by examination of the SC lipid composition. A similar amount of extracted lipids was observed in all FTMs, which was higher in NHS (Figure 3a). The composition of FFAs was determined using the liquid chromatography-mass spectrometry (LC-MS) FFA analysis (Supplementary Figure S2a). The absolute amount of FFAs remained similar despite the reduction in supplemented PA (Figure 3b). As compared to NHS, a substantial reduction in level of FFAs was observed. The reduction in absolute amount of FFAs is mainly ascribed to the reduced presence of saFFAs with a carbon (C) chain length ≥ C23:0 (Figure 3c). The absolute amount of short chain monounsaturated FFA (muFFA) C16:1 and C18:1 was similar in FTMs and NHS, whereas the long chain muFFAs ≥ C22:1 were more abundant in FTMs (Figure 3d). The polyunsaturated FFA (puFFA) C18:2 was substantially reduced in FTMs as compared to NHS (Figure 3e).

The absolute quantities were used to calculate the relative amount of FFAs. No difference in the relative saFFA and muFFA composition was observed between the FTM_100%PA_, FTM_10%PA_, and FTM_1%PA_. When comparing the FTMs with NHS, differences in saFFA and muFFA composition were observed (Figure 3f,g). In contrast to NHS, in the SC of FTMs there was a higher relative level of FFA C20:0 and C22:0, which was counterbalanced by a reduced relative abundance of FFA with chain length ≥ C25:0. The FFA C24:0 remained the most abundant FFA in the SC of both FTMs and of NHS. Regarding the relative abundance of muFFAs, in the SC of FTMs there was a higher level of C20:1, C22:1, and C24:1, which reduced the relative abundance of C18:1, in agreement with the observations of the absolute level of muFFAs (Figure 3g).

The differences between the FFA composition of NHS and FTMs were also reflected in the level of muFFAs and in the MCL of FFAs. A considerable increase in muFFA content in the SC was detected in FTMs as compared to NHS (Figure 3h). In addition, the mean carbon chain length (MCL) of the total FFAs was reduced in FTMs as compared to NHS (Figure 3i). 

Our quantified LC-MS FFA analysis did not include FFA C16:0 and C18:0 due to manufacturer’s contamination of C16:0 and C18:0 in the solvents, and it excluded very long chain muFFAs C26:1 and C28:1 due to the absence of these FFA standards. To still provide an indication on the presence of these FFAs, we analyzed the area under curve (AUC) of the FFAs and corrected these for the internal standard (ISTD) and contamination, followed by plotting the relative amount. First, we observed FFA C16:0 at a similar and C18:0 at a higher level in the FTMs as compared to NHS (Supplementary Figure S3a). Second, we observed that the muFFAs C26:1 and C28:1 are present at higher fractions in the SC of FTMs as compared to that of NHS (Supplementary Figure S3b), in line with previous observations [11]. This indicates that there are more short chain FFAs present in FTMs and it implies that the level of muFFAs calculated with the quantified FFA data is underestimating the fraction of muFFAs in the SC of FTMs.

These results indicate that variations in supplemented PA lead to a similar FFA composition, and that there are substantial differences between the FFA profiles of NHS and FTMs.

### 2.4. Variations in Supplemented PA did not alter the Composition of CERs in the SC

**Figure 4:**
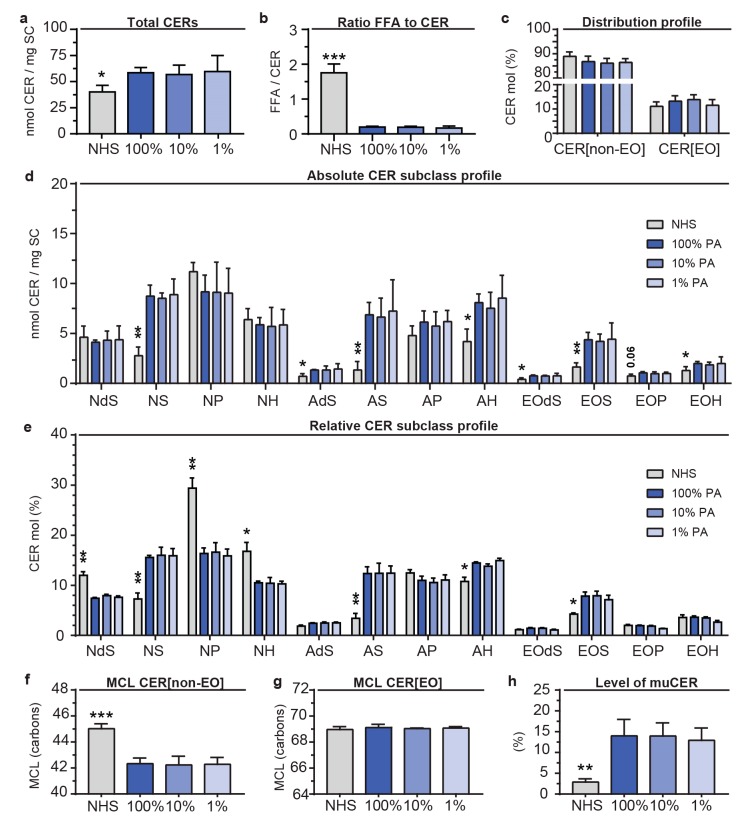
Ceramide (CER) composition in the SC of NHS and in FTM supplemented with different PA levels. (**a**) Absolute amount of CERs in the SC of NHS and of FTMs. (**b**) Ratio of FFAs and CERs present in the SC. (**c**) Distribution profile of total CER[non-EO] and CER[EO] in the SC presented as relative amount. (**d**) Absolute subclass profile of CERs plotted as nmol per mg of SC. (**e**) Subclass profile of CERs plotted as relative amount per CER subclass in percentage of the total CER quantity. (**f**) The MCL of the CER[non-EO] subclasses. (**g**) The MCL of the CER[EO] subclasses. (**h**) The level of muCER in the CER[non-EO] subclasses, presented of total CER[non-EO]. All data for NHS, FTM_100%PA_, FTM_10%PA_, and FTM_1%PA_ represents mean + SD, *n* = 3. Significance indicated by * *p* < 0.05, ** *p* < 0.01, *** *p* < 0.001.

As FFAs serve as building blocks for more complex lipids (i.e., CERs), we assessed whether reduction in PA could affect the CER composition. The CER lipidomic analysis revealed the presence of all major subclasses in NHS and the FTMs on the LC-MS ion maps (Supplementary Figure S2b). In FTMs, an increased presence of CERs with a mass lower than 600 atomic mass units (amu) was detected, which is a premature indication for an altered CER composition. The CER data was quantified to absolute amounts per mg of SC. This revealed a similar absolute amount of CERs in all FTMs, although the level of CER per mg of SC in NHS was significantly lower (Figure 4a). Since the absolute amount of FFA per mg of SC was also calculated (Figure 3b), this provided the opportunity to calculate the ratio between FFAs and CERs at this detailed level. The obtained ratio was drastically reduced in FTMs as compared to NHS, irrespective of PA level (Figure 4b). Then, the composition of the CER subclasses was evaluated by comparing the distribution profile of CER[non-EO] versus CER[EO], which revealed a high level of similarity in all FTMs and NHS (Figure 4c). 

Next, the absolute amount of each CER subclass was determined for FTM_100%PA_, FTM_10%PA_, FTM_1%PA_, and NHS (Figure 4d). The data for CER[non-EO] represents the saturated CER (saCER) and the monounsaturated CER (muCER). The bars of CER[EO] subclasses represent a combination of 4 different subgroups, which are muCER[EO-18:2], saCER[EO-18:2] + muCER[EO-18:1], and saCER[EO-18:1]. The CER subclass profiles of FTMs generated with different PA levels showed a high level of similarity. Considerable differences were observed comparing the subclass profiles of NHS and FTMs, which included an increased quantity of CER[NS], CER [AS], CER [AH], and most of the CER[EO] subclasses. This resulted in a relative abundance of the CER subclasses with a strong reduced fraction of CER [NP], while most other CER subclasses changed in abundancy, but to a lesser extent (Figure 4e). The data strongly indicate that biosynthesis pathways generating CER subclasses [NS], [AS], and [EOS] are most altered in vitro, whereas this is not influenced by PA supplementation.

The CER[EO] subclasses are composed of four different subgroups, as shown in Supplementary Figure S4. In FTMs, no differences were observed upon reduced PA supplementation in the CER[EO] subgroups. Comparing FTMs to NHS, a difference for the CER[EO] subgroups is the presence of saCER[EO-18:1] in contrast to its absence in NHS. 

Subsequently, we addressed the MCL of CER[non-EO] and CER[EO] (Figure 4f,g). The MCL of CER[non-EO] in NHS was higher than that of FTMs, but no differences were observed regarding PA levels. Figure 4g shows a similar MCL of CER[EO] in NHS and FTMs with different PA conditions. The carbon chain length distributions for the CER[non-EO] and CER[EO] deviated significantly from that of NHS, but were similar for the various levels of PA (Supplementary Figure S5). Importantly, the increased presence of C34, C36, C38, C40, and C42 CER[non-EO] in both absolute and relative level in FTMs as compared to NHS is a major difference.

As a final point, the level of muCERs was calculated as percentage of the total CERs (Figure 4h). The level of muCER[non-EO] in the SC of FTMs was not influenced after reduction of the PA concentration, although this was considerably increased in FTMs as compared to NHS. 

### 2.5. FTMs Supplemented with Reduced PA Exhibited a Similar Lipid Organization

**Figure 5:**
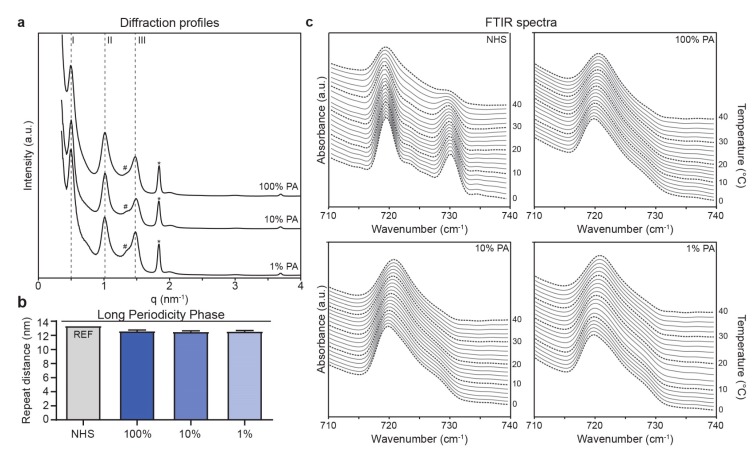
Lipid matrix organization in FTMs supplemented with different PA levels. (**a**) Small angle X-ray diffraction (SAXD) profiles of FTMs plotted as intensity in arbitrary units (a.u.) over the scattering vector q. The orders of diffraction (I, II, and III) are indicated by dashed lines, phase separated cholesterol is indicated by the asterisk (*) symbol. Unknown phases are indicated by the number sign (#). (**b**) Bar plot showing the repeat distance of the LPP in NHS and FTMs based on the indicated orders of diffraction. Data for NHS was obtained from Bouwstra et al. [28], data represents mean + SD, *n* = 3. (**c**) Fourier transform infrared spectroscopy (FTIR) spectra plotted as absorbance over the wavenumber in the methylene rocking vibrational region. On the right y-axis the temperature is shown at which the measurement occurred. Representative FTIR spectra were plotted for NHS and for FTMs with indicated level of supplemented PA.

After addressing the lipid composition, the barrier formation was examined for lamellar organization by small angle X-ray diffraction (SAXD) and for lateral organization by Fourier transform infrared spectroscopy (FTIR). The obtained SAXD diffraction profiles were indicative for the presence of the long periodicity phase (LPP) in FTMs (Figure 5a). The repeat distance of the LPP was determined based on indicated order of diffraction peaks, which revealed a high similarity between FTM_100%PA_, FTM_10%PA_, and FTM_1%PA_ (Figure 5b). The repeat distance of the LPP in FTMs was considerably reduced as compared to that of NHS [28]. For the lateral organization, the FTIR spectrum at the methylene rocking vibration region is indicative for the orthorhombic lateral packing when there were two peaks present, whereas the hexagonal lateral packing is indicated by a single peak [7]. In FTM_100%PA_, FTM_10%PA_, and FTM_1%PA_ the hexagonal lateral packing was observed (Figure 5c). In contrast, the lipids were predominantly arranged in the orthorhombic lateral packing in NHS. Between 30 °C and 40 °C, the intensity of the peak at 730 cm^−1^ reduced in the FTIR spectrum of NHS, indicating a transition from orthorhombic to hexagonal packing.

### 2.6. Reduction of Supplemented PA Resulted in a Similar Expression of Lipid Processing Enzymes

**Figure 6:**
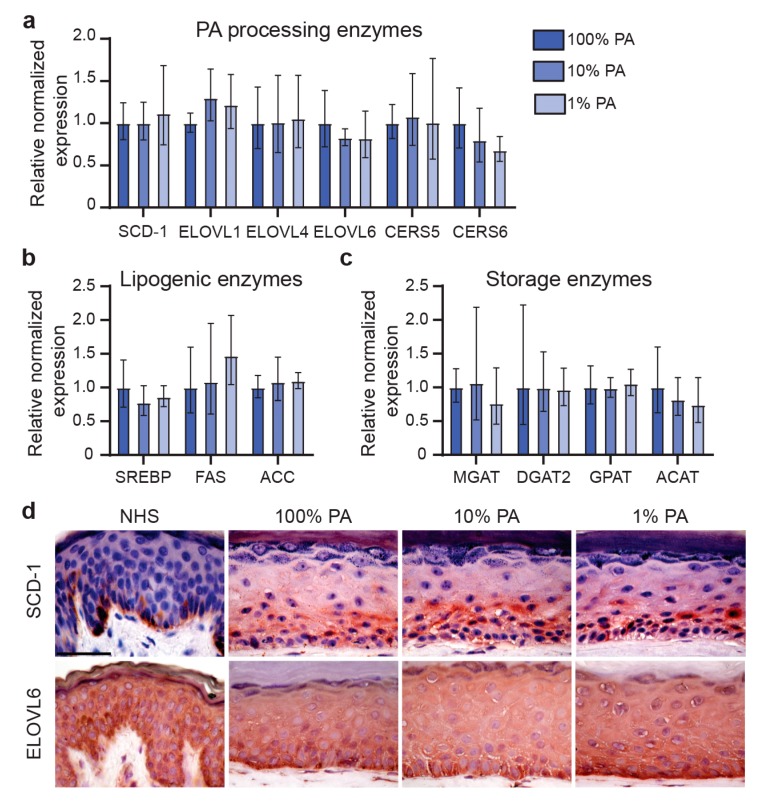
Lipid processing enzyme expression in FTMs supplemented with different PA levels. (**a**) Gene expression of direct and indirect PA processing enzymes *SCD-1*, *ELOVL1*, *ELOVL4*, *ELOVL6*, *CERS5*, and *CERS6* in FTMs supplemented with indicated PA levels. (**b**) Gene expression of lipogenic mediators *SREBP-1c*, *FAS*, and *ACC* in FTMs. (**c**) Gene expression of lipid storage enzymes *MGAT*, *DGAT2*, *GPAT*, and *ACAT* in FTMs. Data represents mean ± SD, *n =* 4. (**d**) Protein expression of PA processing enzymes SCD-1 and ELOVL6 in NHS and indicated FTMs. Proteins are shown in red and nuclei in blue. Scale bar represent 50 μm.

To obtain more information on the lipid biosynthesis pathway in FTMs, we assessed the expression of various lipid processing enzymes related to the barrier formation in the SC. The enzymes *SCD-1*, *ELOVL6*, *CERS5*, and *CERS6* are able to process PA by desaturation to form C16:1, elongation to produce C18:0, or synthesis of CERs with a C16 acyl-chain, respectively [29,30,31]. The level of gene expression for these enzymes was similar in FTM_100%PA_, FTM_10%PA_, and FTM_1%PA_ (Figure 6a). Also, expression of *ELOVL1* and *ELOVL4* remained similar. Furthermore, gene expression of lipogenic mediators (i.e., *SREBP-1c*, *FAS*, *ACC*) remained unaffected (Figure 6b). Lipid storage pathways were unaffected in all conditions tested based on the similar expression of enzymes involved in mono-, di-, and triglyceride synthesis (i.e., *MGAT*, *DGAT2*, *GPAT*) and biosynthesis of cholesterol esters (*ACAT*) (Figure 6c). Additionally, there was no evidence of a change in the amount of diglycerides based on the LC-MS CER ion maps. On protein level, the expression of lipid processing enzymes SCD-1 and ELOVL6 was similar irrespective of PA level supplemented into the medium (Figure 6d). As compared to NHS, the expression of SCD-1 was upregulated in FTMs, whereas the expression of ELOVL6 was similar. Together, these results show that lipid biosynthesis in FTMs remained similar after reduction of supplemented PA.

## 3. Discussion

In this study, we showed that addition of the bioactive lipid PA to the culture medium of HSEs supports a well-orchestrated epidermal morphogenesis. Irrespective of PA level in the culture medium, the SC lipid barrier composition and organization were similar. This indicates that the original level of PA was adequate and that the aberrant lipid barrier formation in HSEs is not associated with the overabundance of this saFFA in the culture medium. Furthermore, we showed that the level of FFAs in the SC of FTMs is substantially reduced as compared to NHS, which emerged as target for future optimization approaches.

Supplementation of a mixture of essential and non-essential lipids to cultured skin substitutes was for the first time implemented by Boyce and Williams [22], who showed that it induced the formation of lamellar bodies and presence of acyl-glucosylceramides to mimic the lipid processing of NHS more closely. Importantly, the medium supplemented FFAs were found to be processed by the epidermis and incorporated into the SC of HSEs [19,32]. Histological assessment revealed similar appearance of the FTMs with varying levels of PA, consistent with results by Spiekstra et al. [33]. However, in the present study we showed that PA has a direct and pronounced effect on epidermal morphogenesis by promoting the proper execution of the early differentiation program and reducing the epidermal activation process. Especially the latter is considered important for a proper barrier formation, as HSEs which display an activated phenotype generally contain a reduced barrier function [7,10]. In line with these observations, in vivo data also suggests that an activated epidermis is associated with a reduced formation and function of the human skin barrier [34,35].

Although epidermal morphogenesis clearly benefits from a sufficient amount of supplemented PA, the gene and/or protein expression of lipid processing enzymes (i.e., elongases, desaturases, or ceramide synthases) was not affected by ten to hundredfold reduction of PA. Furthermore, we obtained insufficient evidence that lipogenic pathways (mediated by *SREBP-1c*, *FAS*, and *ACC*) and lipid storage pathways (mediated by *MGAT*, *DGAT2*, *GPAT*, and *ACAT*) were altered after reduced supplementation of PA. Therefore, it remains of interest to obtain mechanistic insight in the metabolism of external derived FFAs and in de novo lipid biosynthesis of FFAs in the skin. For instance, PA can be processed into ceramides, diacylglycerols, triacylglycerols, or saturated glycolipids (i.e., lyso-phosphatidic acids, lysophosphatidylethanolamine, and phosphatidic acids), although how this affects the SC lipid composition remains to be identified [36]. Otherwise, PA can be utilized for palmitoylation of skin specific proteins, which affect the differentiation and cornification within the epidermis [37].

Other attempts to modulate the in vitro biosynthesis of barrier lipids by changing supplementation of FFAs were performed by Thakoersing et al. [19]. In the absence of PA, no consistent epidermal architecture was observed, while after a fourfold increase in PA supplementation in epidermal models, no strong differences were observed in the composition of barrier lipids. These results suggest that biosynthesis of barrier lipids is difficult to fine-tune by external supplementation of PA, although a basal supplementation level is crucial to support the proper development of FTMs. Reasonably, the dynamic de novo lipid biosynthesis plays a dominant role in this, although determination of the interactions between de novo synthesis and uptake of lipids in vitro remains highly complex due to the dynamic nature of these intertwined processes [38,39,40,41,42]. Alternatively, addition of other classes of bioactive lipids besides FFAs (i.e., phytosphingosine) in the culture medium is a promising approach to improve the SC ceramide subclass profile [43,44].

The altered composition of the major lipids in the SC of HSEs highly contributed to the formation of the aberrant lipid organization, which are in line with previous reports [7,11]. The reduced repeat distance of the LPP could be ascribed to a disbalance in the CER subclass profile in combination with the reduced MCL of both FFAs and CERs [45]. The absence of the SPP is also ascribed to the increased presence of the CER[EO] subclasses, which play a crucial role in the formation of the LPP [46,47]. The lateral organization in HSEs is hexagonal, which is caused by the increased presence of muFFAs and muCERs, the drastic reduction in ratio of FFAs to CERs, and the reduced MCL of both FFAs and CERs, as revealed by lipid membrane models [48,49]. Importantly, these alterations in lipid organization are associated with reduced barrier functionality, which has been confirmed by inside-out and outside-in evaluations [7,10].

Based on the combination of the LC-MS FFA with the LC-MS CER analysis, we were able to calculate the ratio of FFA to CER in the SC of NHS and FTMs. Limiting factor in the FFA analysis was the contamination of the solvents, which forced the exclusion of C16:0 and C18:0. However, after correcting for the impurities, it was indicated that the short chain C18:0 is relatively more present in FTMs than in NHS. Furthermore, the C26:1 and C28:1 were excluded due to a lack of corresponding FFA standards for quantification. However, our results indicate that these muFFAs are more present in FTMs than in NHS. These factors partially contributed to the reduced abundance of FFAs in FTMs. Nonetheless, the absolute quantity as well as the FFA profile in the SC of FTMs emerged as highly interesting and crucial targets for future studies.

Due to the high resolution of our LC-MS CER analysis, increased presence of saCER[EO-18:1] was monitored in the FTMs, which are not present in NHS [50]. The incorporation of oleic acid (C18:1) instead of LA (C18:2) could be a result of reduced availability of the essential FFA C18:2, reduced release from triglycerides, or an excessive rapidity of biosynthesis of CER[EO] during in vitro biosynthesis [20]. Vičanová et al. [20] reported that the addition of 30 µg/mL LA resulted in normalization of the LA content of the SC barrier. Optimization of the concentration of LA in our culture system (8.42 µg/mL) is therefore an interesting strategy to reduce the content of saCER[EO-18:1] and increase the level of saCER[EO-18:2] and the level of puFFA C18:2. On the other hand, LA is a potent ligand for the peroxisome proliferator-activated receptor alpha [51]. Activation of this nuclear receptor induced a substantial increase in the presence of CERs in a skin equivalent model [52]. As this potentially further reduces the FFA to CER ratio, this could be unbeneficial for the lipid composition, organization and barrier functionality. Alternatively, kinetic studies of the essential FFAs added to keratinocyte cultures show that the conversion of LA to AA is extremely rapid [53], emphasizing the dynamic FFA metabolism in keratinocytes and complexity of correcting this in vitro.

With this study, we showed that supplementation with adequate levels of PA during in vitro HSE reconstruction promotes the generation of a correct epidermal morphology, although the lipid barrier formation is similar after a ten to hundredfold reduction in external PA levels, although the lipid barrier formation is similar after a ten to hundredfold reduction in external PA levels. Moreover, there is a substantial reduction in the quantitative presence of FFAs in the SC of HSEs. These findings broaden our understanding on the mechanisms underlying in vitro epidermal morphogenesis and barrier formation and indicate new strategies to better mimic the native skin tissue by HSEs.

## 4. Materials and Methods

### 4.1. Generation of FTMs

Surplus female breast skin tissues were obtained after mamma reduction surgeries according to declaration of Helsinki principles and are regarded in this study as NHS. As reported previously [54], experiments were conducted in accordance with the Dutch law on medical treatment agreement article 7:467 and in accordance with the code for proper use of human tissue of the Dutch federation of biomedical scientific societies. As a consequence of this legislation, it is allowed to use anonymized skin when no objection was made by the well-informed healthy donor.

Epidermis and dermis were separated after removal of adipose tissue followed by enzymatic digestion and primary cell suspensions were obtained and cultured as described before [3,55]. Isolated primary keratinocytes and fibroblasts were tested for mycoplasma contamination using polymerase chain reaction (PCR) analysis before further use. Generation of full thickness models occurred as described before [10]. In short, a dermis was formed by a collagen lattice harboring 1.2 × 10^5^ primary fibroblasts. An epidermis was generated on top of the collagen lattice by seeding 2.5 × 10^5^ primary keratinocytes, which self-organized to form structured epidermal layers after air-exposure. Except for the PA content, this was reduced to 10% and 1% of the original 100% level (25 μM). FFA mixtures were coupled to the carrier molecule bovine serum albumin before supplementation to the medium. FTMs were grown for 14 days at the air-liquid interface in a cell culture incubator (Memmert, Schwabach, Germany) at 37 °C, 90% RH, 7.3% CO_2_, and atmospheric oxygen levels. HSE batches were generated from four different primary cell donors.

### 4.2. Immunohistochemical Analyses

Sections of NHS, FTM_100%_, FTM_10%_, and FTM_1%_ were 24 h fixated in 4% formaldehyde before rehydration and paraffin embedding or snap frozen for cryopreservation. HE staining was performed according to methods provided by the manufacturer (VWR, Breda, The Netherlands). Immunohistochemistry or indirect immunofluorescence was performed on 5 μm sliced formalin fixed paraffin embedded (FFPE) sections. After deparaffinization and rehydration, heat mediated antigen retrieval in citrate buffer (pH 6) was performed. Antigen retrieval for collagen type IV staining was mediated by protease incubation. Next, non-specific antibody binding was reduced by incubation with normal human serum (Sanquin, Leiden, The Netherlands) prior to application of primary antibodies (Table S1). Stainings were performed using the streptavidin–biotin–peroxidase system (GE Healthcare, Buckinghamshire, United Kingdom) according to the manufacturer’s instructions or using indirect immunofluorescence, as described before [10]. Visualization of the sections occurred using a Zeiss Axioplan 2 light microscope (Carl Zeiss BV, Breda, The Netherlands) or a Leica CTR5000 fluorescence microscope (Leica, Wetzlar, Germany). Neither non-specific nor background staining of secondary antibodies were detected (Supplementary Figure S6a). The number of corneocyte layers in the SC was determined by safranin red staining and potassium hydroxide expansion as described before [10,56] (Supplementary Figure S6b). Estimations of the epidermal thickness and proliferation index were performed as reported earlier [10]. Quantification of K1 and K10 positive area occurred using ImageJ software (National Institutes of Health, Bethesda, Maryland, USA). The areas were measured using an in-house developed macro. The non-stained protein areas (no K1 or K10 present) minus the basal layer area was divided with the total epidermis area and was subtracted from 100%.

### 4.3. Gene Expression Analyses

Total RNA was extracted from the viable epidermis of FTMs or epidermis and partially attached dermis of NHS using the Favorprep tissue total RNA mini kit (Favorgen, Ping-Tung, Taiwan). Methods provided by the manufacturer were followed, with a single exception of an additional 15 min DNA digestion step after loading the RNA, by means of a RNAse-free DNAse set (Qiagen, Hilden, Germany). After elution, the RNA concentration was determined using a Nanodrop™ UV-VIS spectrophotometer (Thermofisher, Waltham, MA, USA). Synthesis of complementary DNA and performance of quantitative real-time polymerase chain reactions occurred consistently with methods as described before [57]. Details on primer sequences are provided as Table S2.

### 4.4. Lipidomics Analyses

#### 4.4.1. Lipid Extraction

The stratum corneum was isolated using trypsin digestion and was stored until use in an inert environment as described before [58]. The lipid extraction was performed using an adapted Bligh and Dyer extraction procedure as reported by Boiten et al. [13]. After extraction, samples were stored under argon gas at 4 °C until FFA and CER analyses were performed. Dry weight of SC was determined before and after lipid extraction using a microbalance.

#### 4.4.2. FFA Analysis

Compositional analysis by LC-MS FFA analysis occurred as described before [58]. In short, 2 µL of 0.75 mg/mL lipid extract was injected into a Waters Acquity UPLC H-class system (Waters, Milford, MA, USA) for lipid separation using a Purospher Star LiChroCART reverse phase column with 3 µm particle size and 55 × 2 mm i.d. (Merck, Darmstadt, Germany). Detection occurred by a XEVO TQ-S mass spectrometer (Waters) measuring in negative ion mode between m/z 200 and 550 amu. Data analysis was performed using Masslynx software. Several FFA entities were selected for analysis, including saFFAs (C20:0, C22:0, C24:0, C26:0, C28:0, and C30:0), muFFAs (C16:1, C18:1, C20:1, C22:1, and C24:1), and puFFA (C18:2). Quantification occurred using the AUC by correction for the ISTD deuterated C24 (Cambridge Isotope Laboratories, Andover, MA, USA) and for response using the calibration curves of the FFA standards (C16:1, C18:1, C18:2, C20:0, C20:1, C22:0, C22:1, C24:0, C24:1, C26:0, C28:0, and C30:0) (Sigma-Aldrich, St. Louis, MO, USA). Due to manufacturer’s contamination of C16:0 and C18:0 in the solvents and the lack of available long chain muFFA standards, no additional FFAs were quantified.

#### 4.4.3. CER Analysis

The extracted samples were evaporated under a stream of nitrogen at 40 °C and reconstituted in 95:2½:2½ (*v*/*v*/*v*) heptane:chloroform:methanol to a concentration of 0.3 mg/mL. After addition of the ISTD N(24deuterated)S(18) (Evonik Industries, Essen, Germany), 5 µL of the sample was injected into the UPLC-MS setup. This consisted of an Acquity UPLC H-class coupled to an XEVO TQ-S mass spectrometer (Waters) with an atmospheric pressure chemical ionization chamber. Detection occurred in positive ion mode measuring full scan m/z between 350 and 1200 amu. Separation of CERs was performed on a PVA-Sil column with 5 µm particles size and 100 × 2.1 mm i.d. (YMC, Kyoto, Japan) as described in detail before [13]. Data analysis was performed with Masslynx software. For the composition analysis, 12 CER subclasses were included with nomenclature as described by Motta et al. [59] (Figure S1g). For the CER[non-EO], both saturated and monounsaturated classes were included of: [NdS], [NS], [NP], [NH], [AdS], [AS], [AP], and [AH]. For the CER[EO], both saturated and monounsaturated classes were included of [EOdS], [EOS], [EOP], and [EOH]. In addition, besides the linoleic acid residue (C18:2) also the oleic acid residue (C18:1) in CER[EO] was included as described by Helder et al. [60]. Quantification of the AUCs was performed using a three-dimensional response model according to the processing method described by Boiten et al. [13]. Corrections for the isotope overlap of the CER entities containing two ^13^C atoms to those which only contain ^12^C (resulting in equal mass) were performed. After the corrections, the data were converted to absolute amount per mg SC.

### 4.5. Small Angle X-Ray Diffraction

SAXD analyses were performed with isolated SC. Measurements were performed at the European Synchrotron Radiation Facility at station BM26B as described extensively by Mojumdar et al. [49]. The scattering intensity I was measured as a function of the scattering vector q. The latter is defined as $q=\frac{4\cdot \pi \cdot \sin\theta }{\lambda }$, where *θ* is the scattering angle and where *λ* is the wavelength. Using the peak position (*q_n_*), the repeat distance was calculated using the equation $d=\frac{2n\cdot \pi }{qn}$, where n is the order of diffraction peak. The length of the repeat unit of the lipid lamellae was determined based on the peak position of the first, second and third order of diffraction peak.

### 4.6. Fourier Transform Infrared Spectroscopy

The isolated SC of NHS, FTM_100%PA_, FTM_10%PA_, and FTM_1%PA_ were hydrated during 24 h before placement between two AgBr cells. FTIR signals were acquired on a Varian 670-IR spectrometer (Agilent Technologies, Santa Clara, CA, USA) equipped with a broad-band mercury cadmium telluride detector as described before [57]. The spectrometer was cooled with liquid nitrogen and connected to a temperature regulating device. Data were collected with a frequency range of 400–4000 cm^−1^. Spectral parameters were: Speed 25 kHz, under sampling ratio 2, filter 6.4, and aperture 1 cm^−1^. Data acquisition occurred in a time resolution of 240 sec and a length of run equal to 164 min, resulting in a 1 °C temperature increase per measurement between 0 °C and 40 °C. The spectra were analyzed and deconvoluted with Varian Resolutions Pro software.

### 4.7. Statistics

Statistical analyses were performed with GraphPad Prism software (GraphPad Software, La Jolla, CA, USA). Statistical testing was performed with 1-way or 2-way ANOVA with Holm-Sidak post-test tests between all FTMs. NHS versus FTM_100%PA_ was tested using unpaired student’s t-test. Significant differences are presented as * for *p* < 0.05, ** for *p* < 0.01, or *** for *p* < 0.001 with lines between bars for the former and vertically above NHS for the latter comparison.

## Supplementary Materials

Supplementary materials can be found at https://www.mdpi.com/1422-0067/20/23/6069/s1. Table S1: Antibodies utilized during immunohistochemical or immunofluorescence analyses. Table S2: Primer sequences utilized during qPCR analyses. Figure S1: Intercorneocyte lipid matrix of the SC with main lipid organizations and constituents. Figure S2: LC-MS ion maps of detected FFAs and CERs present in the SC of NHS and of FTMs generated with 100% PA level as most representative map. Figure S3: Corrected peak areas for the composition of FFAs. Figure S4. Compositional analysis of CER[EO] subgroups in the SC of FTMs generated with various PA levels and of NHS. Figure S5. Carbon chain length distribution of CERs in the SC of NHS and of FTMs generated with varying PA levels. Figure S6: Controls of immunohistochemistry and safranin red staining.

## Abbreviations

SC	Stratum corneum
NHS	Native human skin
HSE	Human skin equivalent
FFA	Free fatty acid
CER	Ceramide
LPP	Long periodicity phase
SPP	Short periodicity phase
LA	Linoleic acid
AA	Arachidonic acid
PA	Palmitic acid
SCD-1	Stearoyl-CoA desaturase-1
FTM	Full thickness model
HE	Hematoxylin and eosin
saFFA	Saturated free fatty acid
muFFA	Monounsaturated free fatty acid
puFFA	Polyunsaturated free fatty acid
MCL	Mean carbon chain length
AUC	Area under curve
ISTD	Internal standard
LOQ	Limit of quantification
amu	Atomic mass unit
saCER	Saturated ceramide
muCER	Monounsaturated ceramide
SAXD	Small angle X-ray diffraction
FTIR	Fourier transform infrared spectroscopy
*ELOVL*	Elongation of very long chain fatty acids protein
*CERS*	Ceramide synthase
*SREBP*	Sterol regulatory element-binding protein
*FAS*	Fatty acid synthase
*ACC*	Acetyl-CoA carboxylase
*MGAT*	Monoacyglycerol acyltransferases
*DGAT*	Diacylglycerol acyltransferases
*GPAT*	Glycerol phosphate acyltransferase
*ACAT*	Acyl-coenzyme A:cholesterol acyltransferase
LC-MS	Liquid chromatography-mass spectrometry
UPLC	Ultra performance liquid chromatography
FFPE	Formalin fixed paraffin embedded
