# Supplementary material for: Contribution of Palmitic Acid to Epidermal Morphogenesis and Lipid Barrier Formation in Human Skin Equivalents
Source: Int J Mol Sci. 2019 Dec 2;20(23):6069. doi: 10.3390/ijms20236069 (PMC6928966; doi:10.3390/ijms20236069)
Supplement: Supplementary file 1 [file ijms-20-06069-s001.pdf]

International Journal of *Molecular Sciences*

Contribution of Palmitic Acid to Epidermal Morphogenesis and Lipid Barrier Formation in Human Skin Equivalents

Arnout Mieremet [1,†], Richard Helder [2,†], Andreea Nadaban [2], Gert Gooris [2], Walter Boiten [2], Abdoelwaheb El Ghalbzouri [1,‡] and Joke A. Bouwstra [2,‡,*]

[1] Department of Dermatology, Leiden University Medical Centre, 2333 ZA Leiden, the Netherlands
[2] Division of BioTherapeutics, LACDR, Leiden University, 2333 CC Leiden, the Netherlands
* Correspondence: bouwstra@lacdr.leidenuniv.nl; Tel.: +31-71-527-4208
† These authors contributed equally to this work
‡ Joint senior authorship

Supplementary material

Table S1. Antibodies utilized during immunohistochemical or immunofluorescence analyses.

Target	Material	Host	Clone	Dilution	Supplier
Ki67	FFPE	Mouse	MIB1	1:100	DAKO, Denmark
Cytokeratin 10	FFPE	Mouse	DE-K10	1:50	Labvision/Neomarker, USA
Cytokeratin 1	FFPE	Rabbit	AF87	1:75	Covance, The Netherlands
Cytokeratin 5/8	FFPE	Mouse	RCK201	1:40	Monosan, The Netherlands
Involucrin	FFPE	Mouse	SY5	1:1200	Sanbio, The Netherlands
Cytokeratin 16	FFPE	Mouse	LL025	1:100	Serotec, UK
Cytokeratin 17	FFPE	Mouse	E3	1:1500	EMD Millipore Corporation, USA
Loricrin	FFPE	Rabbit	AF62	1:1000	Covance, The Netherlands
Filaggrin	FFPE	Rabbit	PRB417	1:1000	Covance, The Netherlands
Collagen type IV	FFPE	Mouse	24.12.8 (PHM-12)	1:75	Chemicon, Australia
Laminin 332	Frozen	Mouse	BM165	1:150	Provided by Dr. M. Aumailley, Germany
Vimentin	FFPE	Mouse	V9	1:250	Sigma-Aldrich, Germany
Alpha smooth muscle actin	FFPE	Mouse	1A4	1:500	Abcam, UK
SCD-1	FFPE	Mouse	CD.E10	1:300	Abcam, UK
ELOVL6	FFPE	Rabbit	Poly	1:500	Abcam, UK

Table S2. Primer sequences utilized during qPCR analyses.

Target	Sequence Forward (5′-> 3′)	Sequence Reverse (5′-> 3′)	Amplicon size
*SDHA**	AACCAAACGCTGGGGAAGAA	GGAACACGGCAGCATGATTT	126
*ZNF410**	GCTGTGGTAAGCAGTTTACTACAG	CTTGGGCTTCACAAAGGAAAGG	90
*ARCP2**	TCCGGGACTACCTGCACTAC	GGTTCAGCACCTTGAGGAAG	96
SCD-1	ACAGTGCTGCCCACCTCTTCG	CCCTCACCCACAGCTCCAAGTG	94
ELOVL1	GGAGCTCCAGGTATTGCCAAGG	AGCCGTGGTCCCTGTAGAGCA	203
ELOVL4	TTTGGTGGAAACGATACCTGA	TTAGAGCCCAGTGCATCCAT	120
ELOVL6	TCGGTGCTCTTCGAACTGGTGC	GTATCTCCTAGTTCGGGTGCTTTGC	154
CERS5	CGGCTCTGTGACACCCTTT	TCTCAAAGAGGGTCGTGTTCA	100
CERS6	TGTTTGTTATGTTTGCCGTGGT	CCAACGATCTCCCAGCTTTCA	100
SREBP-1c	GGAGGGGTAGGGCCAACGGCCT	CATGTCTTCGAAAGTGCAATCC	80
FAS	CATCCCCACCTATGGCCTGC	GCCTGATGCAGTCGATGTAGTA	89
ACC	CATATCCAGTCCATGCTGCGT	GTTCCAGCCACTGCACAACC	100
MGAT	GCAGAGAGCAGGTGTGTGAT	GCCTCCTCTGGACTATGGGA	71
DGAT2	GCAAGGGCTTTGTGAAACTGG	CCTCGAAGATCACCTGCTTGTA	99
GPAT	ATGAAACACCAGATGGACGGA	TCCTAGAACGTGTGCCTTCC	120
ACAT	TGCTTCAGGAATGAAAGCCATC	CATCCCACCTGCCACCATC	82

* References

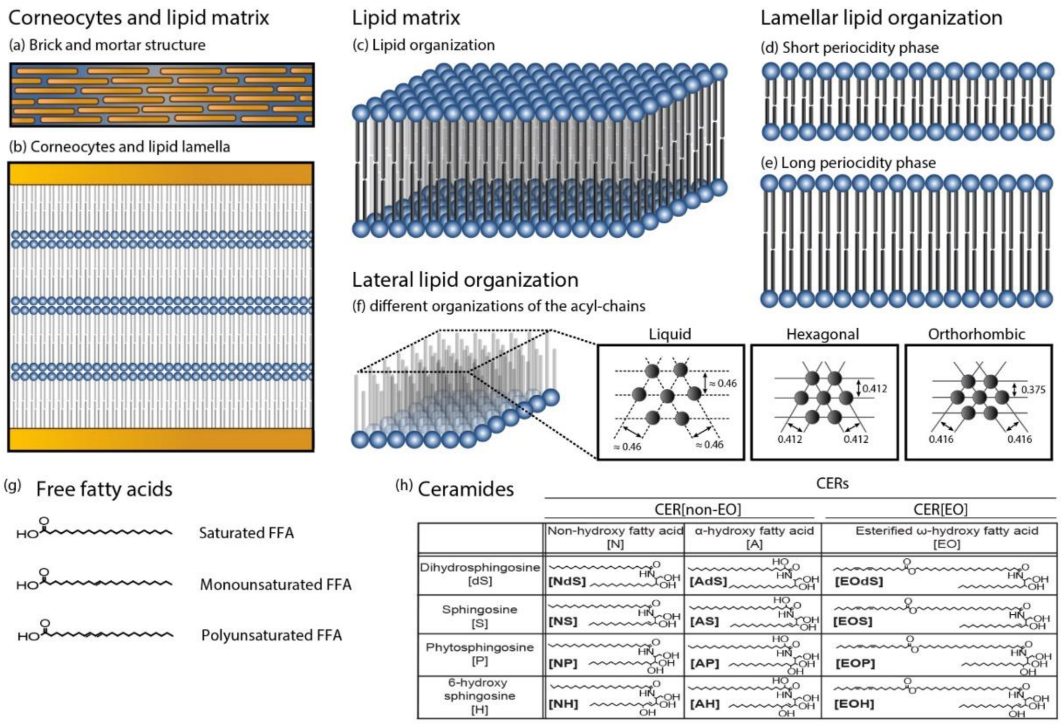

Figure S1. Intercorneocyte lipid matrix of the SC with main lipid organizations and constituents. (**a**) Simplified schematic overview of the SC which figuratively considered as brick and mortar structure. (**b**) Isolated elements of the lipid matrix with repetitive pattern as detected in the intercorneocyte space. (**c**) Schematic overview of the lipid molecular arrangement, with head groups as dark blue spheres and hydrocarbon chains in dark grey. (**d**) Lipids in the lamellar organization shown in the short periodicity phase with a repeat distance of approximately 6 nm [1]. (**e**) Lipids in the lamellar organization shown in the long periodicity phase with a repeat distance of approximately 13 nm [1]. (**f**) Lateral organization of the hydrocarbon chains, which adopt the liquid, hexagonal, or orthorhombic organization. Approximations of the intermolecular space for each organization is indicated. Images were adapted from Helder et al. [2]. (**g**) Schematic overview of the FFA subgroups analyzed in this study. (**h**) Tabular overview of the 12 CER subclasses analyzed in this study with nomenclature according to Motta et al. [3].

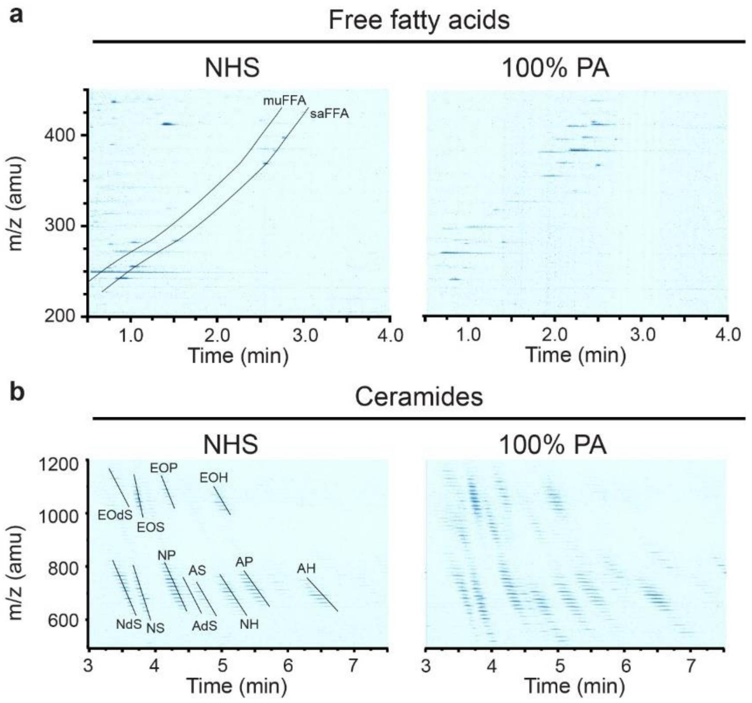

Figure S2. LC-MS ion maps of detected FFAs and CERs present in the SC of NHS and of FTMs generated with 100% PA level as most representative map. (**a**) LC-MS FFA ion maps with position of saFFAs and muFFAs indicated in the plot of NHS. (**b**) LC-MS CER ion maps with indicated position and names of 12 CER subclasses according to Motta et al. [3] in the plot of NHS.

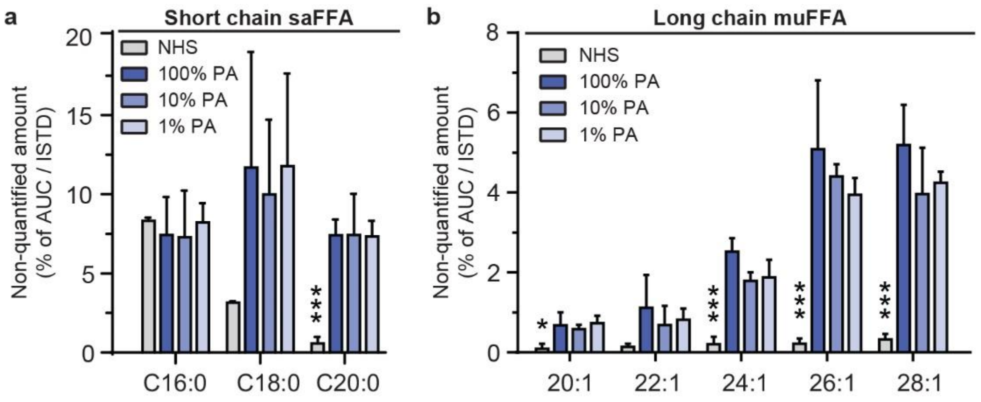

Figure S3. Corrected peak areas for the composition of FFAs. (**a**) Relative level of short chain saFFAs calculated as AUC divided by ISTD and corrected for solvent impurities. Data is presented in relative peak areas as percentage of the total AUC / ISTD of saFFA C16:0 – C30:0. (**b**) Relative level of long chain muFFAs calculated as AUC divided by ISTD. Data is presented in relative peak areas as percentage of the total AUC / ISTD of muFFA C16:1 – muFFA C28:1. All data is provided for NHS and for FTMs with indicated level of supplemented PA. Data indicates mean + SD, $n = 3$. Significant differences are noted as *, **, and *** corresponding to $P < 0.05$, <0.01, and <0.001.

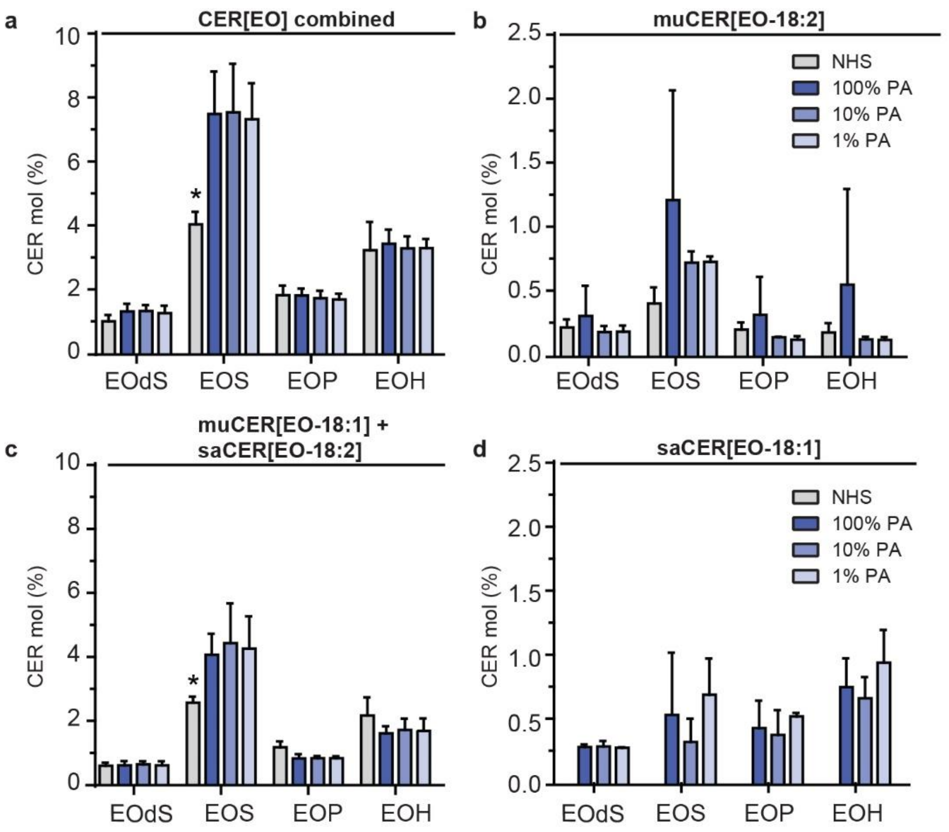

Figure S4. Compositional analysis of CER[EO] subgroups in the SC of FTMs generated with various PA levels and of NHS. (**a**) Relative level in of total CER[EO] plotted per subclass, which is the cumulative amount of muCER[EO-18:2], saCER[EO-18:2] + muCER[EO-18:1], and saCER[EO-18:1]. (**b**) Relative level of muCER[EO-18:2] plotted per subclass. (**c**) Relative level of saCER[EO-18:2] + muCER[EO-18:1]. (**d**) Relative level of saCER[EO-18:1]. All data is provided for NHS and for FTMs with indicated level of supplemented PA. Data indicates mean + SD, $n = 3$. Significant differences are noted as *, **, and *** corresponding to $p < 0.05$, <0.01, and <0.001.

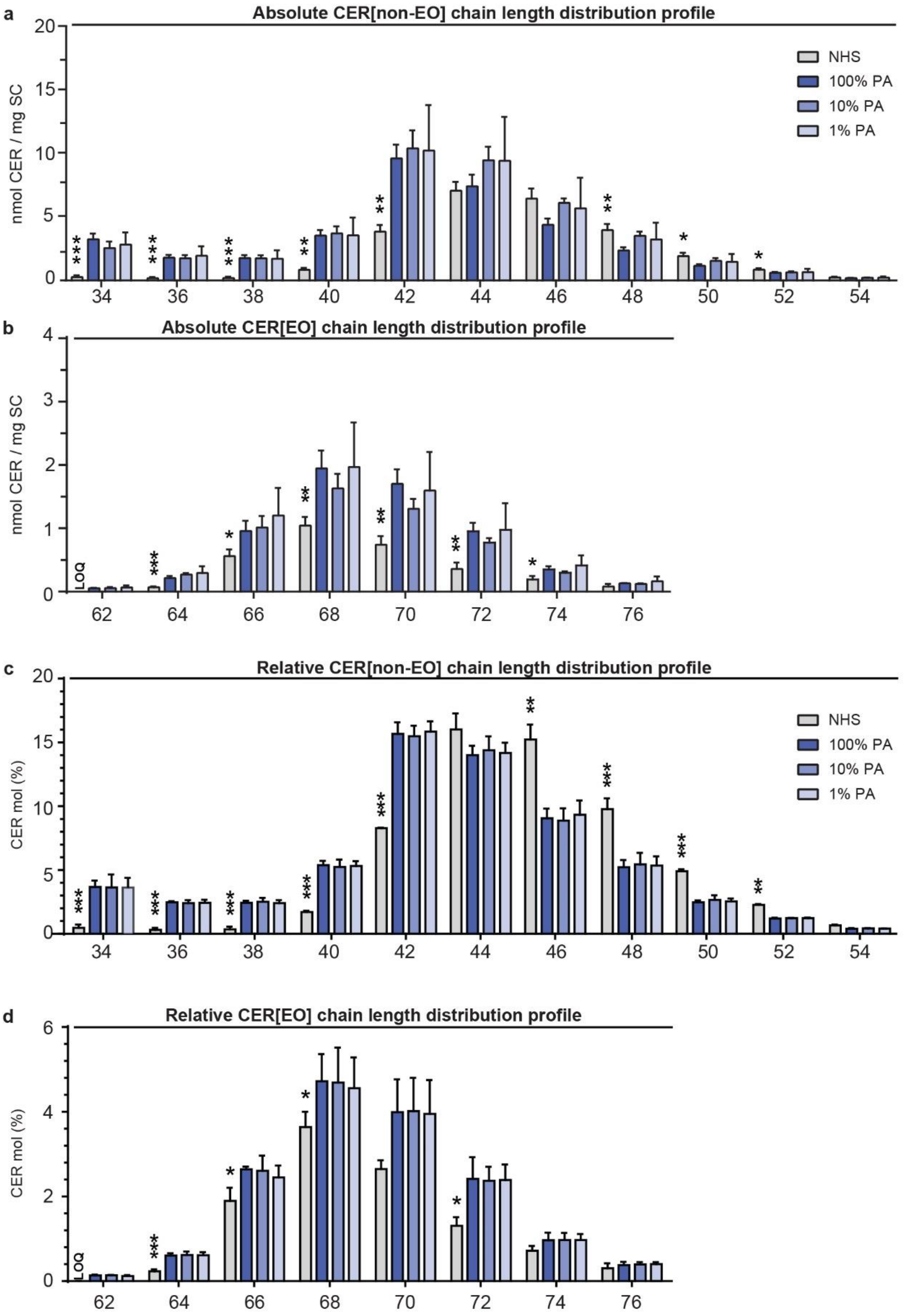

Figure S5. Carbon chain length distribution of CERs in the SC of NHS and of FTMs generated with varying PA levels. (**a**) Bar diagram plot of absolute level of CER[non-EO] with an even number of carbon atoms. (**b**) The absolute level of CER[EO] with an even number of carbon atoms. (**c**) Bar diagram plot of relative level of CER[non-EO] with an even number of carbon atoms (**d**) The relative level CER[EO] with an even number of carbon atoms. Data is shown for NHS, $FTM_{100\%PA}$, $FTM_{10\%PA}$, and $FTM_{1\%PA}$ and represents mean + SD, $n = 3$. LOQ indicated below limit of quantification. Significant differences are noted as *, **, and *** corresponding to $p < 0.05$, <0.01, and <0.001.

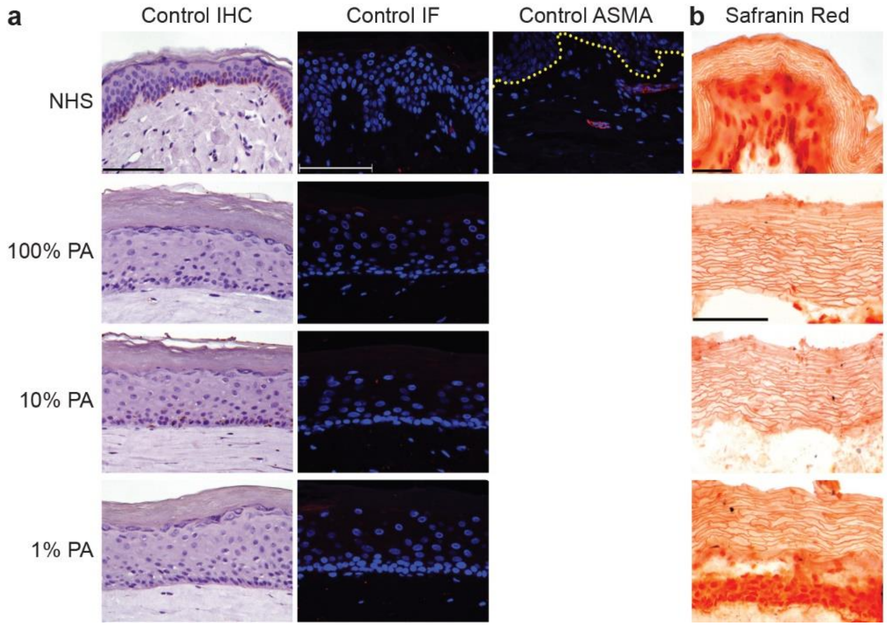

Figure S6. Controls of immunohistochemistry and safranin red staining. (**a**) Negative controls for immunohistochemical (IHC) and immunofluorescence (IF) stainings. Positive control for ASMA staining in NHS. Nuclei are stained blue using hematoxylin or DAPI, yellow dotted line indicates dermal-epidermal junction. (**b**) Representative cross sections of NHS and FTMs stained with safranin red followed by potassium hydroxide expansion of the SC used for quantification of the number of corneocyte layers. Scale bar indicates 100 µm.

Supplementary References

1. Bouwstra, J. A.; Gooris, G. S.; van der Spek, J. A.; Bras, W., Structural Investigations of Human Stratum Corneum by Small-Angle X-Ray Scattering. *J. Investig. Dermatol.* **1991,** *97*, 1005–1012.
2. Helder, R. W. J.; Boiten, W. A.; van Dijk, R.; Gooris, G. S.; El Ghalbzouri, A.; Bouwstra, J. A., The effects of LXR agonist T0901317 and LXR antagonist GSK2033 on morphogenesis and lipid properties in full thickness skin models. *Biochim. Et Biophys. Acta* **2020**, *1865*, 158564..
3. Motta, S.; Monti, M.; Sesana, S.; Caputo, R.; Carelli, S.; Ghidoni, R., Ceramide composition of the psoriatic scale. *Biochim. Et Biophys. Acta* **1993,** *1182*, 147–151.
